# PSMD14 is a novel prognostic marker and therapeutic target in osteosarcoma

**DOI:** 10.1186/s13000-024-01489-y

**Published:** 2024-06-11

**Authors:** Jiabin Lai, Weike Kong, Qiangchang Fu, Zhaochang Jiang, Bohao Sun, Xin Ye, Jing Kong, Shumei Wei, Lifeng Jiang

**Affiliations:** 1 Department of Orthopedic Surgery, The Second Affiliated Hospital, Zhejiang University School of Medicine, Hangzhou, China; 2 Key Laboratory of Motor System Disease Research and Precision Therapy of Zhejiang Province, Hangzhou, China; 3 Department of Pathology, Second Affiliated Hospital, School of Medicine, Zhejiang University, Hangzhou, 310009 China; 4 Department of Endocrinology, The Second Affiliated Hospital, School of Medicine, Zhejiang University, Hangzhou, China; 5 Orthopedics Research Institute of Zhejiang University, Hangzhou, China; 6 Clinical Research Center of Motor System Disease of Zhejiang Province, Hangzhou, China

**Keywords:** Osteosarcoma, PSMD14, Prognosis, Metastasis

## Abstract

**Background:**

Osteosarcoma is a bone tumor that is characterized by high malignancy and a high mortality rate, and that originates from primitive osteoblastic mesenchymal cells and is most common in rapidly growing long bones. PSMD14, also known as RPN11 or POH1, is a member of the JAMM isopeptidase family, which is able to remove the substrate protein ubiquitination label, thereby regulating the stability and function of the substrate protein. In this study, we explored the expression and potential biological significance of the PSMD14 deubiquitinating enzyme in osteosarcoma.

**Methods:**

Immunohistochemical methods were used to detect the expression of PSMD14 in biopsies of 91 osteosarcoma patients, and the specimens were classified into high and low PSMD14 expression groups. The correlation between PSMD14 expression and clinical indicators and prognosis was compared.SiRNA was used to downregulate PSMD14 in two osteosarcoma cell lines (HOS and SJSA-1), and the effects of downregulation of PSMD14 on the viability, proliferation, and invasion ability of osteosarcoma cells were analyzed.

**Results:**

We identified significant differences in recurrence, metastasis, and survival time of the osteosarcoma patients on the basis of PSMD14 expression. High expression of PSMD14 in osteosarcoma patients was associated with a low survival rate and high risk of metastasis and recurrence. Down-regulation of PSMD14 inhibited the viability, proliferation, and invasiveness of osteosarcoma cell lines.

**Conclusions:**

PSMD14 may be a new prognostic marker and therapeutic target for osteosarcoma.

## Background

Osteosarcoma is a malignant bone tumor that mainly affects children and young adults between 10–20 years old, characterized by the direct production of bone or bone-like tissue by proliferating tumor cells and exhibits a high degree of malignancy, strong invasion, rapid disease progression, and high mortality [1, 2]. The current treatment for osteosarcoma includes surgical resection in conjunction with systemic chemotherapy to contain potential micro-metastases [3]. The 5-year event free survival (EFS) of patients with localized osteosarcoma is approximately 70%, while patients with metastatic or recurrent disease fare poorly, with an overall survival rate of less than 20% [4]. Biomarkers for osteosarcoma diagnosis and prediction of prognosis and chemotherapy resistance have not been identified. Thus, the identification of an effective, sensitive, and specific biomarker for the diagnosis and prognosis prediction of osteosarcoma patients is critical to improving patient survival and may also lead to new therapeutic targets for osteosarcoma.

There are two main protein degradation pathways in eukaryotic cells: the lysosomal pathway, which is mainly responsible for the degradation of foreign proteins or membrane proteins that enter cells through endocytosis; and the ubiquitin–proteasome system (UPS) pathway, which is the main mechanism of protein degradation in eukaryotic cells, mediates degradation of old or damaged intracellular proteins [5]. Deubiquitinases (DUBs; also known as deubiquitylating or deubiquitinating enzymes) can remove ubiquitin chains from post-translationally modified proteins, leading to a reversal of ubiquitin signaling or protein stabilization by rescue from either protea-somal(for example, cytosolic proteins) or lysosomal (for example, internalized receptors) degradation [6].

PSMD14, also known as RPN11 or POH1, is a subunit of the regulatory particle of the proteasome that functions as an intrinsic deubiquitinase to remove polyubiquitin chains from substrate proteins [7]. Increasing evidence has indicated that PSMD14 is a key oncogenic factor that promotes tumor growth in several cancers [8]. Bioinformatics analysis showed that high expression of PSMD14 was associated with sarcoma metastasis and prognosis [9]. However, research on the role of PSMD14 in osteosarcoma is limited.

In this study, we explored the expression and potential function of PSMD14 in osteosarcoma. We used immunohistochemical methods to detect PSMD14 expression in osteosarcoma tissues and analyzed the correlation between PSMD14 expression and metastasis, prognosis, and chemotherapy response. We also performed functional studies by knocking down PSMD14 in osteosarcoma cell lines by RNA interference and examining cell proliferation and invasion.

## Methods

### Tissue specimens

A total of 91 paraffin-embedded tissues from the Department of Pathology of the Second Affiliated Hospital of Medical College of Zhejiang University (Binjiang Hospital) from 2015 to 2018 were selected. All patients were diagnosed with abiopsy and received chemotherapy before radical operation; the prognosis of patients was monitored. All specimens were collected with the permission of the Ethics Committee of the Second Affiliated Hospital of Medical College of Zhejiang University and the informed consent of patients.

### Clinicopathological evaluation and follow-up

Complete data were available for all cases. There was no history of serious disease or cancer treatment including radiotherapy and chemotherapy before the biopsy.

Survival time was determined as the number of months from the time the patient was diagnosed by the first biopsy to the date of death or last visit before study completion. At the return visit, 44 patients had metastasis or recurrence during the follow-up period and 41 of these patients had lung metastasis; 47 patients had no metastasis or recurrence.

### Immunohistochemical staining

Tissue samples were fixed in 10% neutral formalin within 30 min after resection and then dehydrated, transparentized, and embedded using routine protocols. Continuous 3-µm-thick sections were heated for 2 h and subjected to immunohistochemical staining using the Leica BOND-III fully automated IHC staining system. The sections were counterstained with hematoxylin, dehydrated with gradient alcohol, transparentized with xylene, and mounted with resin. Samples were stained with rabbit monoclonal PSMD14 antibody (1:1000 in antibody dilution medium, EPR4257; Abcam); secondary antibody staining was performed using the BOND IHC Polymer Detection Kit (antibody dilution medium, DS9800; Leica; Germany).

### Evaluation of immunohistochemical staining

The immunohistochemical results were independently evaluated by two experienced pathologists and scored using Friedrich’ s criteria.

Positive cells were judged as cells with brown granules in the cytoplasm or nucleus. Five high-power microscopic visual fields were selected for each section, and 100 cells in each visual field were examined. The staining intensity of positive tumor cells was scored as follows: 0 (negative), 1 (mild), 2 (moderate), or 3 (strong). The proportion of positive tumor cells was scored as follows: 1 (0%–5%), 2 (6%–50%), 3 (51%–75%), or 4 (76%–100%). The two scores were multiplied to obtain the total score (0–12). Low expression was defined as a total score ≤ 6, and high expression was defined as a score > 6.

### Evaluation of chemotherapy response

The Huvos grading system, which is based on the necrosis rate of tumor tissue in postoperative radical specimens, was used to evaluate the chemotherapy response. Grade I indicates almost no tumor necrosis caused by chemotherapy, grade II indicates mild effectiveness of chemotherapy with a necrosis rate > 50% and some residual viable tumor tissue, grade III indicates partial effectiveness of chemotherapy with a necrosis rate > 90% and some residual tumor tissue visible on some slices, and grade IV indicates no viable tumor tissue observed in all slices.

### Cells and cell culture

The HOS and SJSA-1 human osteosarcoma cell lines were obtained from the Cell Collection of the Chinese Academy of Sciences. Cells were cultured in DMEM containing 10% fetal bovine serum and 1% antibiotic (penicillin and streptomycin) in a cell incubator at 37 °C with 5% CO_2_.

### RNA interference

Osteosarcoma cells were infected with siRNAs against PSMD14, The nucleotide sequences of the siRNAs were as follows: PSMD14-RNAi(114751–2):CAGTGAACATTGTAAACACAA,PSMD14-RNAi(114752–1):CATGGACTAAACAGACATTAT,PSMD14-RNAi(114753–1):CAAGCCATCTATCCAGGCATT. Cell transfection was performed using LipofectamineTM 2000 transfection (Thermo, USA) reagent following the manufacturer’s instructions. HOS and SJSA-1 cells were inoculated into 6-well plates one day before transfection with a cell density of 5 × 105 cells per well. and cultured with a new DMEM medium for two hours before transfection. PSMD14 siRNAs, si-NC, and LipofectamineTM 2000 reagent were diluted with OPTI-MEM medium and incubated for 5 min. Then, PSMD14 siRNA and si-NC were gently mixed with LipofectamineTM 2000 regent and incubated for 20 min at room temperature. The transfection mixture was added to cells. After 6 h, the medium was replaced with DMEM containing 10% serum and cells were returned to the incubator.

### Cell Counting Kit‑8 (CCK‑8) assay

Transfected cells were inoculated into 96-well plates (200 µl, 5000 cells per well) with six wells for each transfection group, and plates were cultured at 37℃ and 5% CO_2_. After 24 h and 48 h, 20 µl CCK-8 reagent was added to each well, and cells were incubated at 37℃ for 1 h. The optical density was measured at 450 nm on a microplate reader. The proliferation activity of cells in different groups was statistically analyzed. The experiment was repeated three times.

### Colony formation assay

Cells were seeded in six-well plates with 500 cells per well. Each transfection group contained three wells, and media were changed every three days. After 14 days of culture, the cells were washed with PBS, fixed with formaldehyde at room temperature for 15 min, and stained with crystal violet for 15 min. Stained cells were counted under an inverted microscope.

### Invasion assays

A Transwell chamber in a 24-well plate was used for cell invasion assays. Matrigel (BD, USA) was coated on the upper surface of the membrane of the chamber. Cell suspensions (200 µL, 2 × 10^5^ cells/mL) were added to the top chamber of the Transwell system; 700 µl of complete medium was added to the bottom chamber. The Transwell chamber was then incubated at 37 °C with 5% CO_2_ for 24 h. Cells that did not penetrate the membrane on the upper layer were removed with a moist cotton swab; the remaining cells were fixed in 4% paraformaldehyde at room temperature for 20 min and stained with crystal violet. After drying, the number of invasive cells was counted in five randomly selected fields under a light microscope. The experiment was repeated three times.

### Western blot analysis

Transfected cells were washed three times with pre-chilled PBS and lysed with RIPA buffer. The protein concentration of cell lysates was determined using a BCA analysis kit. Protein samples (20 µg) were separated by SDS-PAGE and transferred to a PVDF membrane using the wet transfer method. The membrane was blocked with 5% skimmed milk at room temperature for 2 h and incubated with primary antibody overnight at 4℃; after three washes with TBST, the membrane was incubated with secondary antibody at room temperature for 2 h and washed three times with TBST. Bands were detected using ECL. Band intensity was analyzed using Image J; β-actin was used as a loading control. The experiment was repeated three times.

### Real-time PCR

Real-time fluorescence quantitative PCR was used to evaluate PSMD14 mRNA levels in osteosarcoma cell lines transfected with one of the three PSDM14 shRNAs or control siRNAs. Total RNA was extracted from HOS cells and SJSA-1 cells using Trizol following the manufacturer’s instructions. A reverse transcription kit (Takara) was used for reverse transcription. The Takara SYBRGreen (Thermo, USA) kit was used for real-time fluorescence quantitative PCR. The PCR reaction conditions were as follows: pre-denaturation at 95℃ for 10 min, denaturation at 95℃ for 15 s, annealing at 55℃ for 15 s, extension at 72℃ for 15 s, and 40 cyclic reactions. The primers for quantitative PCR were as follows:F:5´-AGTTGGATGGAAGGTTTGACACT-3´,R:5´-GTCCTGCTTGCCAACATTCTTTA-3´. The experiment was repeated three times.

### Statistical analysis

Statistical analysis was performed using SPSS. The relationship between PSMD14 expression and clinicopathological characteristics was analyzed using Chi-squared test. Differences in the results among groups were compared using unpaired t-test or two-way ANOVA test. Survival curves were plotted using the Kaplan–Meier survival test and the log-rank test. A *P* value less than 0.05 indicated statistical significance.

## Results

### PSMD14 expression in osteosarcoma tissues.

**Fig. 1:**
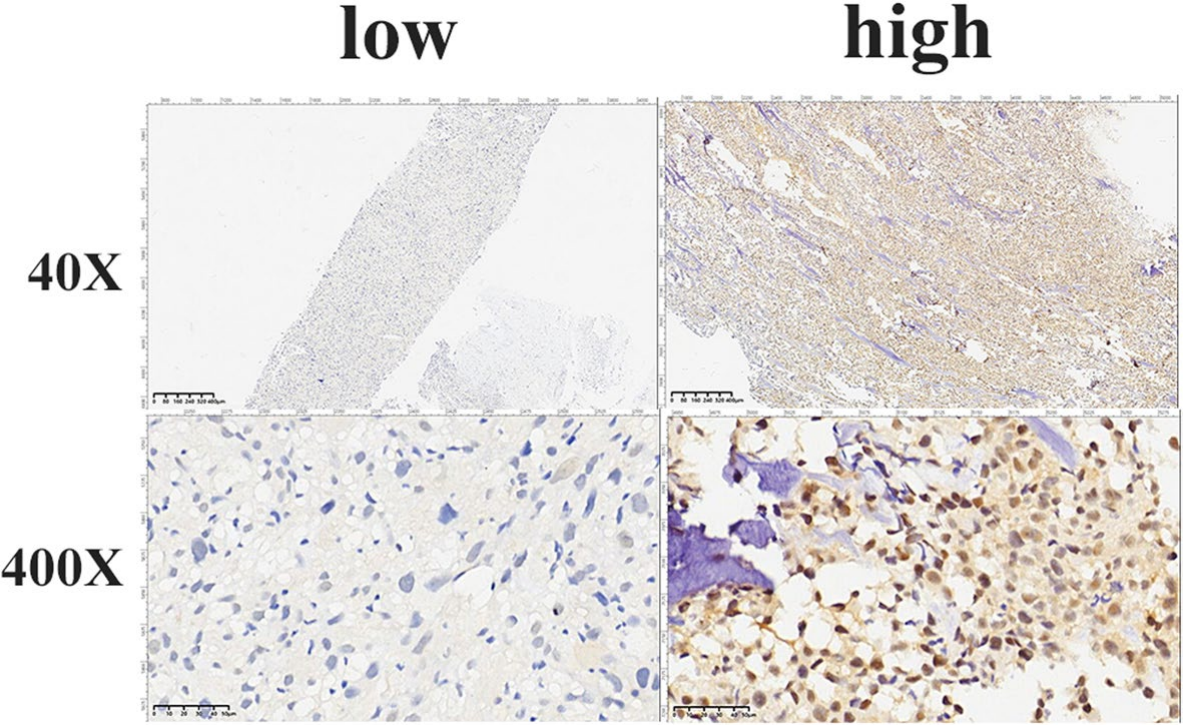
Immunostaining of PSMD14 in osteosarcoma tissues, low expression and high expression

To evaluate the expression of PSMD14 in osteosarcoma, we performed immunohistochemical staining on 91 biopsy specimens from osteosarcoma patients. PSMD14 was detected in the nucleus and cytoplasm of osteosarcoma cells. Expression was scored as described in the Methods. Among the 91 specimens, 72 cases had low PSMD14 expression (72/91), and 19 cases had high PSMD14 expression. As illustrated in Fig. 1.

### Association of the expression of PSMD14 with clinicopathological characteristics in osteosarcoma

**Fig. 2:**
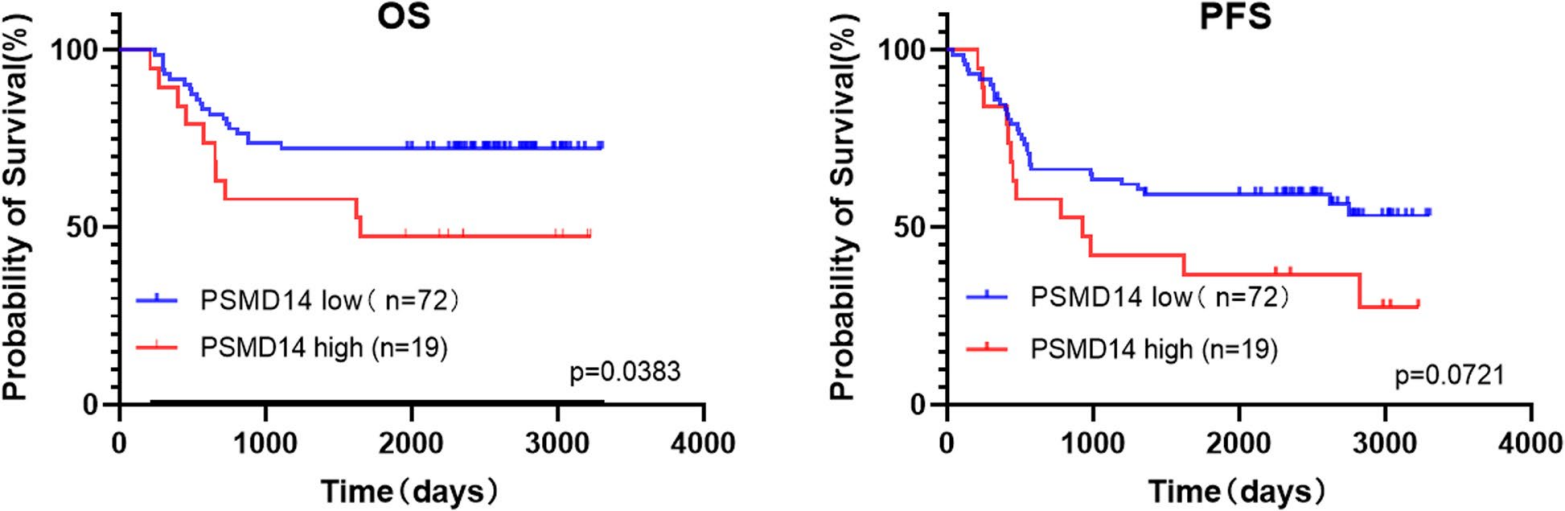
Relationship between PSMD14 expression level and survival in patients with osteosarcoma

There were no significant differences in patient age, sex and tumor location among the PSMD14 expression groups. However, there were significant differences in tumer stage, recurrence and metastasis within five years, and survival time among the two groups (*P* < 0.05). With the increase of PSMD14 expression, the risk of tumour stage, recurrence and metastasis all increased, and the overall survival time decreased. There was no significant difference in the effect of chemotherapy among the PSMD14 expression groups (Table 1 and Fig. 2).

**Table 1:** Relationship between PSMD14 expression level and clinical factors in patients with osteosarcoma

Clinicopathological features	Cases	PSMD14		
		Low	High	*P* value
**Age**				0.8065
≤ 18	69	55	14	
> 18	22	17	5	
**Sex**				0.3118
Male	53	40	13	
Female	38	32	6	
**Position**				0.1872
Femur	56	43	13	
TIBIA	19	15	4	
Other	16	14	2	
**Tumour stage**				0.0462^*^
I, II	76	63	13	
III	15	9	6	
**Metastasis within 5yrs**				0.0491^*^
Yes	44	31	13	
No	47	41	6	
**Survival within 5yrs**				0.0404^*^
Yes	61	52	9	
No	30	20	10	
**Hvous**				0.2217
1, 2	51	38	13	
3, 4	40	34	6	

*P < 0.05

### Knockdown of PSMD14 in osteosarcoma cells reduced the viability, proliferation, and invasion activities of osteosarcoma cells in vitro

To explore the function of PSMD14 in osteosarcoma cells, we selected two different osteosarcoma cell lines, HOS and SJSA-1. We downregulated PSMD14 in osteosarcoma cells using three siRNAs (114751–2, 114752–1, and 114753–1). The siRNA (114751–2) and siRNA (114753–1) were used for subsequent experiments, respectively defined as siPSMD14-1 and siPSMD14-2.

**Fig. 3:**
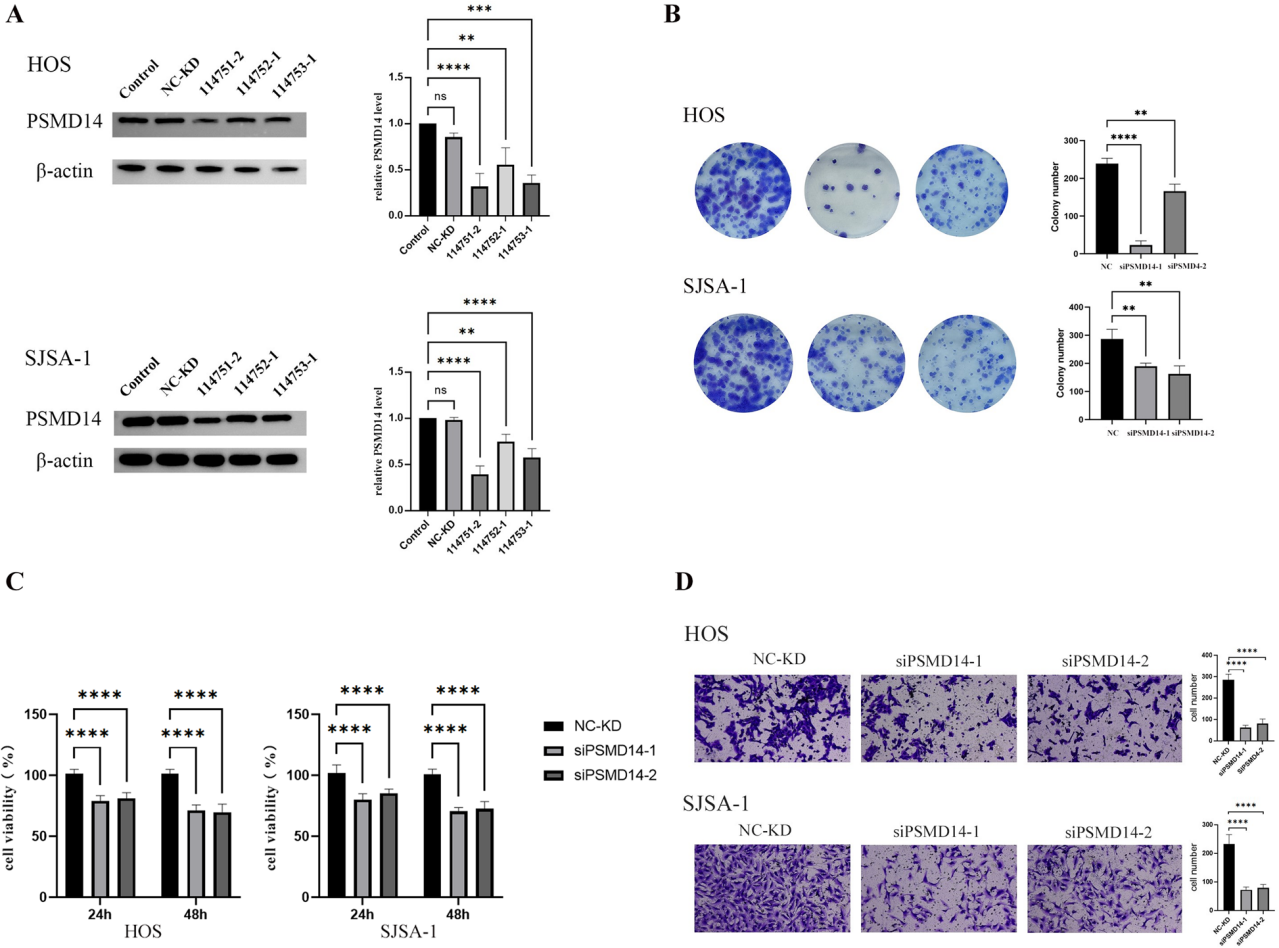
**A** The expression level of PSMD14 was detected by Western blot. **B** HOS and SJSA-1 cell lines were selected for cloning and crystal violet staining with siPSMD14-1(114751–2) and siPSMD14-2(114753–1). **C** The cell viability of knock-down HOS and SJSA-1 cell lines was detected at 24 h and 48 h by CCK-8 assay. **D** the invasive ability of tumor cells was detected by the Transwell test

Colony formation assay and crystal violet staining showed that the proliferation of cells was markedly inhibited after knocking down PSMD14 in the two cell lines (Fig. 3B). We further evaluated the effect of PSMD14 on the cell viability of osteosarcoma cells by CCK-8 and found that cell viability was significantly inhibited with PSMD14 knockdown (Fig. 3C). Transwell experiments revealed that knockdown of PSMD14 significantly inhibited the invasive ability of osteosarcoma cells. These results suggest that PSMD14 may play a role in promoting tumor proliferation and metastasis in osteosarcoma.

## Discussion

Osteosarcomas are bone tumors that are commonly found in pediatric and adolescent patients; these tumors are characterized by a high risk of metastatic progression and recurrence after therapy [1]. Bone metastases are common, and in some cases, lung metastases also develop [10]. While research has focused on diagnosing and treating osteosarcoma, identifying prognostic indicators is also an important area of investigation. However, predicting the prognosis of osteosarcoma patients is complex, and no definitive prognostic factors have been identified.

Bioinformatics analysis suggested that PSMD14 has the potential to be a new biomarker or possible therapeutic target for osteosarcoma [9]. PSMD14 is a 310 amino acid protein encoded by a 12 exon gene located on chromosome 2q24.2 [11]. Studies have shown that PSMD14 can participate in a variety of physiological processes by regulating protein deubiquitination and stability, including differentiation [12], cell viability [13], pluripotency [14], autophagy [15, 16], DNA damage repair [16], immune inflammatory response [17], and so on. In recent years, researchers have shown that PSMD14 affects tumor occurrence and development by regulating multiple malignant biological behaviors such as proliferation, invasion, metastasis, and chemotherapy resistance [8]. In liver cancer, PSMD14 mediates the deubiquitination of GRB2 at lysine residues 48 and 63, thereby increasing GRB2 protein stability and inhibiting degradation. Mutation of the JAMM domain of PSMD14 (C120S and H113Q) or deletion of this domain eliminated the regulation of PSMD14 on GRB2 expression. Aberrant expression of GRB2 activates the Akt, ERK, and STAT3 signaling pathways, promoting liver cancer growth and metastasis [18]. In lung cancer, PSMD14 is significantly upregulated in tumor tissue compared with adjacent normal tissue, and its high expression is positively correlated with tumor size, lymph node metastasis, and TNM staging [19]. Patients with lung adenocarcinoma with high PSMD14 expression have poor OS and DFS. In colorectal cancer, PSMD14 mediates the deubiquitination of ALK2 to enhance its stability and activate the BMP6 signaling pathway. The ALK2-PSMD14 axis also plays an important role in the occurrence of colorectal cancer and cancer stem cells [20].

In this study, we investigated the role of PSMD14 in osteosarcoma. We found that knocking down PSMD14 expression in two osteosarcoma cell lines, HOS and SJSA-1, significantly inhibited cell proliferation and invasion, consistent with previous studies in lung, colorectal, and prostate cancer. Additionally, immunohistochemical results showed that PSMD14 expression correlated with clinical pathological parameters, including overall survival and tumor transfer, suggesting that increased PSMD14 expression is associated with poor prognosis. This finding may provide some guidance for the clinical evaluation of patient prognosis.

Our results did not show a correlation between PSMD14 expression and chemotherapy response. In previous studies using mammalian cell experiments, the results showed that the PSMD14 gene was resistant not only to astrosporin but also to anticancer drugs such as paclitaxel, adriamycin, cisplatin, melphalan, and vincristine [21]. Knocking down the expression of PSMD14 in colorectal cancer reduced the number of tumor cells and drug resistance [20]; however, the mechanism of PSMD14 in drug resistance has not been fully elucidated. The mechanism of chemotherapy resistance to osteosarcoma is not clear and may involve changes in DNA topoisomerase activity, the increase of glutathione transferase activity, membrane transport dysfunction, autophagy activation, or enhanced DNA damage repair [21]. As osteosarcoma is a rare disease, the insufficient study sample size is also an important reason to limit our research results, especially in the study of tumor chemotherapy response. The mechanism of PSMD14 response to chemotherapy in osteosarcoma will be our next research focus, and we hope to collect more patient data to verify the role of PSMD14 in the response to chemotherapy in osteosarcoma.

Several recently developed PSMD14 (POH1) inhibitors, such as capzimin and thiolutin, have shown good anticancer activity in solid tumors and leukemia cell lines [22, 23]. The PSMD14 inhibitor capzimin inhibits the growth of prostate cancer cells and enhances the inhibitory effects of androgen deprivation and docetaxel [23]. In head and neck squamous cell carcinoma and esophageal squamous cell carcinoma, thiolutin can Significantly inhibit tumor growth and increase its sensitivity to cisplatin [24, 25].

Pharmacological inhibition of PSMD14(Rpn11) with O-phenanthroline (OPA) blocks cellular proteasome function, induces apoptosis in MM cells, and overcomes resistance to proteasome inhibitor bortezomib [26]. In addition, OPA was able to inhibit the proliferation, colony formation, motility, and invasion of HCC cells in vitro and the growth and distant metastasis of HCC cells in vivo [18]. A number of studies reported that targeting PSMD14 inhibits tumor growth and migration through SMAD3 accumulation or SLUG decrease in melanoma [27]. These results suggest the potential clinical significance of targeting PSMD14 in solid tumors. Therefore, future studies should explore the possibility of targeting PSMD14 as a new strategy for the treatment of osteosarcoma.

## Conclusion

Our study suggests that PSMD14 may serve as a potential biomarker or therapeutic target for osteosarcoma. Further research is needed to elucidate the precise mechanism by which PSMD14 regulates osteosarcoma progression and to evaluate the potential of PSMD14 as a prognostic indicator and therapeutic target in clinical practice.

## Data Availability

No datasets were generated or analysed during the current study.
